# Coral reef attributes associated with microplastic exposure

**DOI:** 10.1007/s00338-024-02596-4

**Published:** 2025-02-01

**Authors:** Cheryl Hankins, Danielle Lasseigne, Sarah M. Davis, Kimberly Edwards, Jenny S. Paul

**Affiliations:** 1 United States Environmental Protection Agency, Office of Research and Development, Center for Environmental Measurement and Modeling, Gulf Ecosystem Measurement and Modeling Division, Gulf Breeze, FL, USA; 2 United States Environmental Protection Agency, Office of Research and Development, ORISE Research Participation Program, Narragansett, RI, USA; 3 CSS, Inc., under contract to NOAA National Centers for Coastal Ocean Science, Fairfax, VA, USA

**Keywords:** Microfibers, Coral, Biological condition gradient, USVI, Florida

## Abstract

Coral reef declines have been documented since the 1980’s from a variety of global and local stressors. Management resource tools are needed to preserve these fragile ecosystems from stressors, both known and unknown. Laboratory studies have shown microplastics (MP) to have negative effects on coral physiology, but their effects in a natural environment are not well understood. Thus, our primary objectives were to explore associations between MPs and coral reef attributes. We measured MP concentrations from sub-surface water and coral tissue samples from two Caribbean/Atlantic scleractinian coral species, *Montastraea cavernosa* and *Orbicella faveolata*, from St. John, U.S. Virgin Islands and Florida’s Coral Reef in 2017 and 2018, respectively. Polymer identification yielded mostly cotton or polyester fibers for both water and coral tissue samples. This study is the first to document MPs in coral tissues from either the U.S. Virgin Islands or Florida’s Coral Reef and is the first to explore how MPs relate to coral reef attributes. Significant, positive relationships were seen between MPs in coral tissue and coral density, rugosity, and percent coral cover, indicating MPs may not have immediate adverse effects on coral reef health.

## Introduction

Coral reefs contain approximately 25% of the world’s biodiversity and have significance to many indigenous cultures as well as importance for tourism, recreation, and other ecosystem services (Reaka-Kudla 1997; UGML 2007; Knowlton et al. 2010; Gregg et al. 2015). Economically, coral reefs of the United States (U.S.) are estimated to be worth $3.4 billion cumulatively based on several factors including tourism and recreation, coastal protection, amenity, research, and avoided damage costs (Brander and van Beukering 2013).

Coral reefs of the Anthropocene have experienced drastic declines since the 1980s (Gardner et al. 2003; Bruno and Selig 2007; Eddy et al. 2018; Babcock et al. 2020). Global stressors, such as elevated temperatures and ocean acidification, are contributing to the decline of reef habitats (Pastorok and Bilyard 1985; Rogers 1990; Lamb et al. 2018; Eakin et al. 2019; Guan et al. 2020; Cornwall et al. 2021). Additionally, multiple land-use stressors are negatively correlated with coral condition indicators (Smith et al. 2008; Oliver et al. 2011). Coral reefs in Florida (USA) and the U.S. Virgin Islands (USVI), specifically, have been subjected to land-based pollution such as eutrophication and sedimentation (Dustan and Halas 1987; Gray et al. 2008; Edmonds and Gray 2014; Miller et al. 2016; LaPointe et al. 2019) and are currently listed as “impaired” and “fair” condition, respectively (Blondeau et al. 2020; Towle et al. 2020). To protect coral reefs, better understanding of threats to coral health is needed in addition to management tools that focus on both global and local threats (Good and Bahr 2021).

Microplastics (MP), defined as plastic particles < 5 mm in size, are an emerging concern to coral reefs. The presence of MPs is well documented across the globe, even in remote locations (Jamieson et al. 2019; Kanhai et al. 2019; Kelly et al. 2020; Tan et al. 2020; Tekman et al. 2020; Wang et al. 2021; Padha et al. 2022). In a marine environment, MPs can be colonized by microbial and epifaunal growth and can also adhere pollutants (Teuten et al. 2007; Andrady 2011; Zettler et al. 2013). Surface accumulation decreases the buoyancy of MP particles making them more bioavailable to benthic organisms (Ye and Andrady 1991; Teuten et al. 2007), such as scleractinian corals, as has been observed with macroplastics around the globe (Pinheiro et al. 2023). Coral exposure to MPs can affect feeding, stress response, immune system function, coral-host signaling, zooxanthellae photosynthetic efficiency, growth, and has also been linked to bleaching and tissue necrosis (Chapron et al. 2018; Tang et al. 2018; Axworthy and Padilla-Gamiño 2019; Reichert et al. 2019; Syakti et al. 2019; Huang et al. 2021; Lanctôt et al. 2020; Hankins et al. 2021). However, effects of MPs are unclear as most laboratory studies are conducted with spheres or fragments despite most MPs found in reef habitats are fibers (Ding et al. 2019; Patterson et al. 2022), many of which are treated with chemical additives (Athey and Erdle 2022). Furthermore, although MPs can affect individual coral health it is unknown how effects scale up to coral reef assemblages.

The U.S. National Oceanic and Atmospheric Administration (NOAA) conducts surveys for the National Coral Reef Monitoring Program (NCRMP) which provides characterization of fish communities and benthic (i.e., coral reef) habitats. Benthic surveys include measurements of macroinvertebrates, estimates of benthic habitat, and coral demographics (NOAA 2017a, b, NOAA 2018a, b). Line point intercept (LPI) and coral demographic (DEMO) protocols, two approaches used by NCRMP, were used to inform the U.S. Environmental Protection Agency’s (EPA) newly developed coral reef Biological Condition Gradient (Table 1) (Bradley et al. 2020; Santavy et al. 2022a, b).

The coral reef Biological Condition Gradient (hereafter referred to as “BCG”) is an assessment tool developed to support resource managers for protecting or restoring the biological integrity of U.S. waters as mandated by The United States Clean Water Act (CWA; 33 U.S.C. § 1251 et seq. 1972). The development of attributes used in the BCG was created with the assistance of an expert panel of coral reef ecologists (US EPA 2021) to determine the most important characteristics of Caribbean reefs. However, full assessments can be labor intensive and costly; finding an indicator, or a targeted set of indicators, can be a more economical approach to assess habitat condition. In this study, we evaluated the individual BCG attributes obtained from LPI and DEMO NCRMP protocols measured at St. John, U.S. Virgin Islands and Florida’s Coral Reef, to identify significant relationships and importance to concentrations of MPs in water and coral samples.

## Materials and methods

**Fig. 1:**
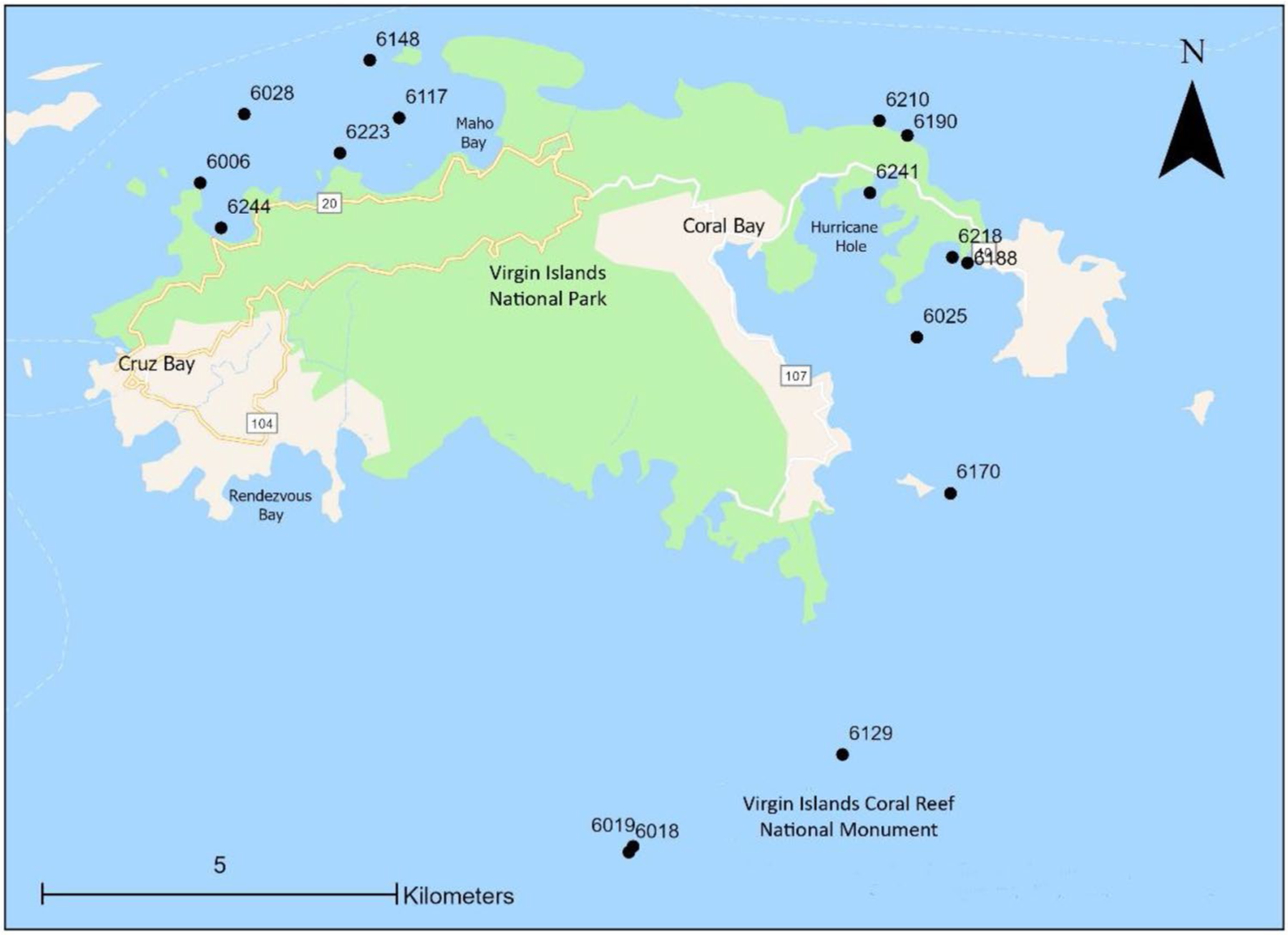
Microplastic sample sites around St. John, USVI surveyed in 2017. Numbers represent site identification designation from the National Oceanographic Atmospheric Administration’s National Coral Reef Monitoring Program survey design. For additional information on sites, see Supplementary Information – A

**Fig. 2:**
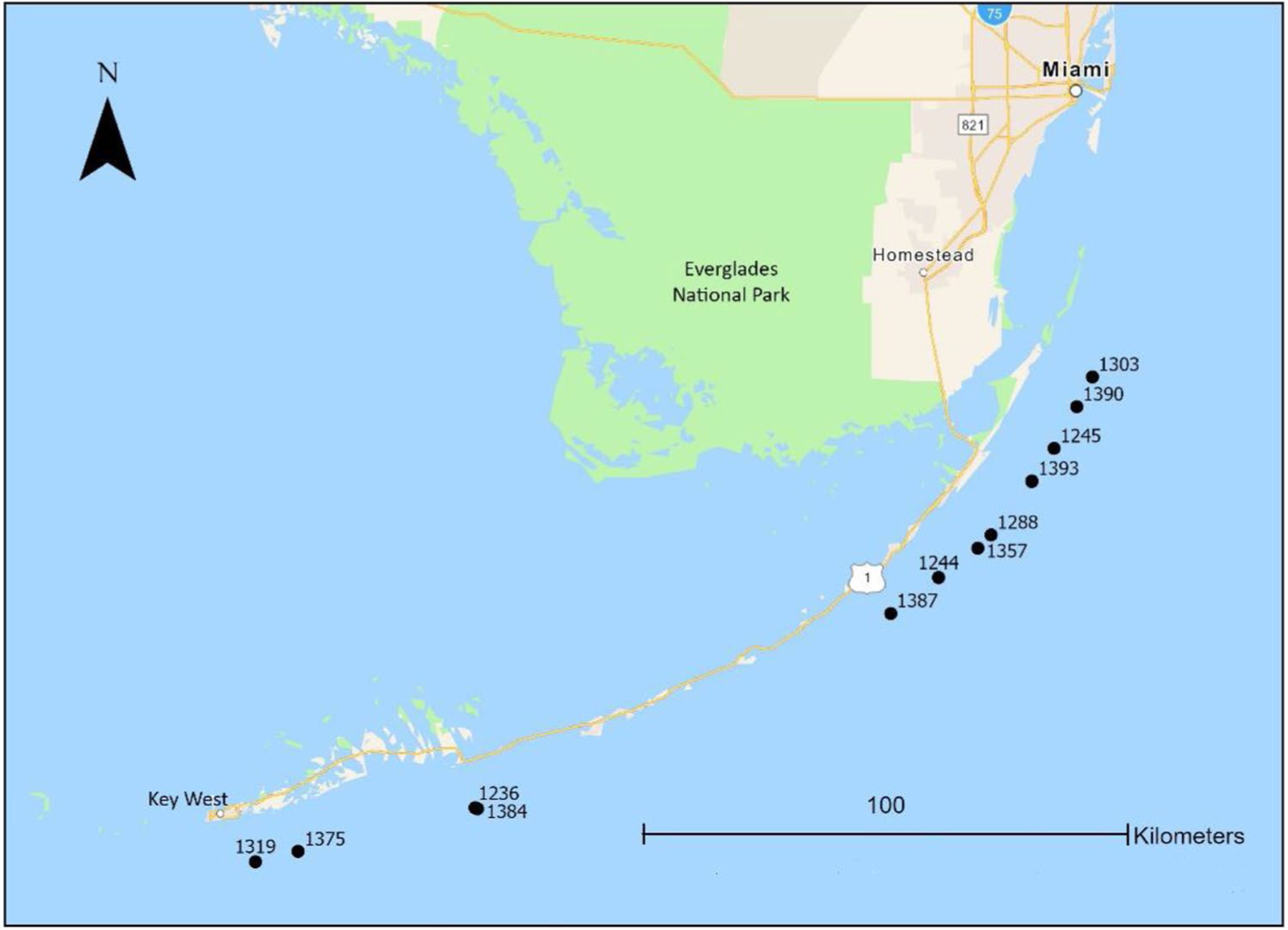
Microplastic sample sites on Florida’s Coral Reef surveyed in 2018. Numbers represent site identifying designation from the National Oceanographic Atmospheric Administration’s National Coral Reef Monitoring Program survey design. For additional information on sites, see Supplementary Information – A

All sampling was conducted concurrently with NOAA’s NCRMP. Water sampling sites (*n*=16) occurred in the vicinity of St. John, USVI on the North and South sides of the island (Fig. 1) in July-August 2017. Sites (*n*=12) on Florida’s Coral Reef were located around Key Largo and Cudjoe Key (Fig. 2) and sampled in August 2018. Coral sampling and benthic surveys (i.e., LPI and DEMO) were also conducted at the same time as water sampling but due to unsafe diving conditions, not all sites have coral tissue or benthic data. Only sites with corresponding water, coral, and benthic data were used for statistical analyses (USVI *n*=12 and FL *n*=7) while summaries of MP particles in water represent data from all sites. GPS coordinates were recorded at each site and water depth was recorded for coral sampling sites (SI-A).

### Environmental water samples

Sub-surface water samples were collected using a submersible bilge pump (Rule^®^ Series 800 Model 20DA). The pump was connected to 10 m of 2.54 cm diameter rigid tubing and a flexible, vinyl coated lead weight was secured around the rigid tubing at the connection of the pump to ensure the pump maintained a depth of approximately 3 m in the water. The tubing was marked every half meter up to 6 m. The bilge pump was operated by a 12 V Sealed Lead Acid battery (Duracell^®^ Model 12-12F2). The bilge pump ran for 1 min prior to the start of collection. After one minute, the effluent of the tubing was placed over a 63-μm sieve (Gilson^®^ sieve #230) for six minutes to filter approximately 100 L of water. The sieve was rinsed with de-ionized (DI) water and the contents retained on the sieve were collected in acid washed glass (473 ml) mason jars (Ball^®^ Model 2,155,752). Upon returning to land, samples were kept in a cool, dark location until transferring to the Coral Research Facility at US EPA’s Gulf Ecosystem Measurement and Modeling Division in Gulf Breeze, Florida where they were stored under refrigeration (4 °C) until processed.

In the laboratory, environmental water samples were transferred from mason jars to 60 mL glass vials filled with no more than 50 mL of sample. Multiple vials were used for each sample. Mason jars were rinsed with Milli-Q^®^ water to ensure all contents were transferred to the vial and rinsate added to vials. Each vial was sonicated using a 750 W ultrasonic processor (Sonics & Materials, Inc. VCX750) with 13 mm converter (Sonics & Materials, Inc. CV334) for 30 s at 30% amplitude to break up organic material (Wagner et al. 2017; Hankins et al. 2018). All vials associated with one sample were poured collectively through a 41-μm nylon filter (Millipore CAT# NY10900) using a 320 mL ceramic Büchner funnel with a rubber stopper using1 L vacuum flask attached to vacuum pump (GAST DOA-P104-AA). During the processing, an inverted 115 mm Pyrex glass Petri dish was used to cover the funnel opening to prevent potential contamination from the air in the laboratory. Once all liquid was removed from the funnel, the nylon filter was transferred to a 110 × 10 mm Pyrex glass Petri dish set, covered, and placed into a drying oven at 55 °C for a minimum of 24 h, after which the Petri dish set containing the filters was placed into a desiccator chamber to cool and prevent atmospheric water absorption onto filter. The filters were stored in the desiccator until quantification.

### Coral tissue samples

Coral colony fragments (~ 13 cm^2^) were collected via SCUBA with a hammer and chisel from sites, provided the target species *Montastraea cavernosa* and *Orbicella faveolata* were present (USVI Permit #s VIIS-2017-SCI-0032 and VICR-2017-SCI-0011, Florida Keys Permit # FKNMS-2017-151) and diving conditions were safe. Up to two coral colony fragments per species were collected from each site. At depth, each coral fragment was placed in an acid washed 120 mL glass jar with a Teflon^®^ coated lid. Jars and lids were acid washed prior to use in the field; however, the interior of the container was exposed to ambient seawater when the coral fragments were placed into the jars at depth. To minimize unintentional sample contamination from ambient seawater collected with the coral fragment, the seawater collected with the coral fragment in the jar was discarded when the samples were brought to the boat at the conclusion of the dive. The entire surface of the coral fragment was then thoroughly rinsed with artificial seawater (35 ppt) made with de-ionized (DI) water, and the interior surfaces of the jar and lid were rinsed with DI water. The coral fragment was placed back in the jar, and the jar was filled with 90% ethanol and 35 ppt artificial seawater in a 1:1 ratio. Coral samples were placed on ice in the field and stored at − 20 °C until shipping. Coral samples were shipped frozen to Gulf Breeze, FL and then stored at 4 °C until analysis.

In the laboratory, coral fragments were removed from jars containing ethanol using metal forceps and rinsed with Milli-Q^®^ water. Rinse water was collected into the jar containing the ethanol. The coral fragment was then placed into a clean 120 mL jar containing a 7.5% hypochlorite solution for 2 h to remove tissue from the skeleton. The sodium hypochlorite solution was filtered through a 5 μm membrane filter (Millipore^®^ TMTP09030) prior to use to remove potential fibers or microplastics that may have been present in the solution from the manufacturing or storage process. If a skeleton fragment still had tissue attached, it remained in sodium hypochlorite solution for an additional hour. Once all tissue was removed from the skeleton, ethanol and sodium hypochlorite were separately transferred into multiple 60 mL glass vials. The 120 mL glass jars that held ethanol and sodium hypochlorite were rinsed with Milli-Q^®^ to ensure all sample transferred, and the rinsate was retained. Vials containing these solutions and dissolved coral tissue were sonicated for 30 s at 35% amplitude to break up organic material. The samples were then filtered using the same process described previously for the environmental water samples using a 41-μm nylon filter (Millipore CAT# NY10900), covering the Büchner funnel during processing, drying the filter for a minimum of 24 h, and storage of the dried filter in a desiccator chamber. The alcohol and the sodium hypochlorite solution were filtered into separate vacuum flasks to prevent the production of chloroform.

Tissue surface area of the fragment was used to standardize the number of microplastics found in each coral fragment for comparisons across sites. The skeleton remaining after tissue removal was used to obtain a three-dimensional scan of the fragment using an Artec^®^ Space Spider 3D scanner (Reichert et al. 2016). The scanner was held approximately 30 cm from the coral roughly at a 30° horizontal angle, while the turntable was rotated 360°. In the same scan, the scanner was moved to an approximate 80° angle, and another rotation completed. Using Artec^®^ Studio software, the skeletal portion that was covered with tissue was selected and the total tissue surface area calculated.

### Quantification and polymer identification of microplastics

Petri dishes containing nylon filters were examined under a stereo microscope at a maximum 4 × magnification (Olympus SZ61) outfitted with a camera (Olympus SC30). The microscope was contained in a ventilated balance safety enclosure to prevent potential sample contamination during the quantification process. A custom-built counting chamber was used to quantify MPs. The counting chamber had an inset grid consisting of sixty-nine 1 cm square cells; some cells were slightly larger but contained at least one side that was 1 cm. The glass Petri dish was labeled at the top for orientation purposes in case a particle needed to be re-examined. The Petri dish fit snuggly on the grid. Visual identification of particles followed Lusher et al. (2020) key for classification of suspected microplastic particles whereby MP morphologies were categorized into one of seven morphologies: fragment, sphere, pellet, fiber, fiber bundle, foam, or film. Particle number, grid cell location, and morphology were recorded. Particles less than 50 μm were excluded (coral samples only) as 99.98% of MP found in coral reef water was greater than 50 μm (Carbery et al. 2022) and has been suggested as the lower observation limit (Dris et al. 2017). Digital photos were taken of each particle observed and cataloged for documentation purposes. Using visual identification methods from Lusher et al. (2020), the type of MP was recorded along with any descriptions, if necessary, and confirmed by an additional reviewer.

A representative subsample of particles (approximately 6%) was selected from each location for material identification of polymer (total particles analyzed, *n* = 30). Particles were mounted onto glass slides using double-sided tape and material types identified using an in Via Qontor Raman Microscope (Renishaw plc., Gloucestershire, UK). Spectra were acquired from a point in the center of each particle under 100 × magnification using a 785-nm excitation laser with 10 s integration time and for 1 accumulation. Laser power was variable from 1 to 10% based on particle color and relative background fluorescence. Spectra were processed using WiRe software (WIRE 5.4) to remove cosmic rays and flatten background fluorescence and then denoised with Savitzky-Golay filter (Araujo et al. 2018). Several spectral libraries were used for matching resulting spectra, including an internal standards library made from known materials, OpenSpecy (Cowager et al. 2021), and SLoPP (spectral library of plastic particles) and SLoPP-E (spectral library of plastic particles ages in the environment) (Munno et al. 2020). Spectral matches > 70% were accepted, with matches between 70 and 40% considered individually by user visual confirmation of relevant functional groups and manual exclusions of identifiable additive peaks (pigments, etc.) (El Khatib et al. 2023).

To control for potential environmental contamination by particulates during sample handling and processing, coral and water samples were processed under a laminar flow hood except during sonication. Additionally, color dyed, 100% cotton laboratory coats were worn by researchers throughout processing to avoid synthetic fiber contamination of the samples. Glassware used during processing was acid washed beforehand using an acid dish washer (Lancer 815 LX Ultima). Prior to processing water samples, Milli-Q water control samples were processed using these handling approaches to ensure there was no environmental contamination introduced to samples from the laboratory.

### Coral reef attributes

Line point intercept (LPI) and coral demographic protocols (DEMO) (NOAA 2017a, b, NOAA 2018a, b) were applied in the field using SCUBA. Using reef condition metrics utilized by the BCG, as well as weighted rugosity from LPI, the resulting descriptive coral reef benthic habitat data (Table 1) were compiled. Weighted rugosity was calculated based on the frequency of one of six binned height classes in each meter section along the LPI transect.

### Data analysis: Microplastics and coral reef attributes

Statistical analysis for MP data and coral reef BCG attributes was limited to sites with corresponding water, coral, and benthic data. All statistical tests were performed using R (V 4.3.1; R core team 2020). Site 6188 from USVI was removed from statistical analysis after first suspected as an outlier by exploratory PCA and confirmed via Grubbs tests on individual reef attributes (*α* = 0.05) (*outliers* V 0.15; Komsta 2022). Differences in tissue MP concentrations between species were assessed via one-way ANOVA with sampling location as random effect (*lme4* V 1.1.34; Bates et al. 2015). Differences in MP concentrations (water and tissue) BCG attributes between sampling locations were assessed via Wilcoxon non-parametric t tests. Linear mixed effects models were used to evaluate relationships between MP in tissue and water depth, water MP concentrations, and individual reef attributes parameters with sampling location set as random effect and significance (*α* = 0.05) determined by log-likelihood tests (*lme4* V 1.1.34; Bates et al. 2015). A principal components analysis (PCA) was performed on all variables (centered and scaled) (*vegan* V 2.6.4; Oksanen et al. 2022). To quantify relationships between PCA variables, a Spearman correlation matrix was constructed and evaluated for significant *p* values (*α* = 0.05) (*psych* V 2.4.3; Revelle 2024).

## Results

### Environmental water samples

**Fig. 3:**
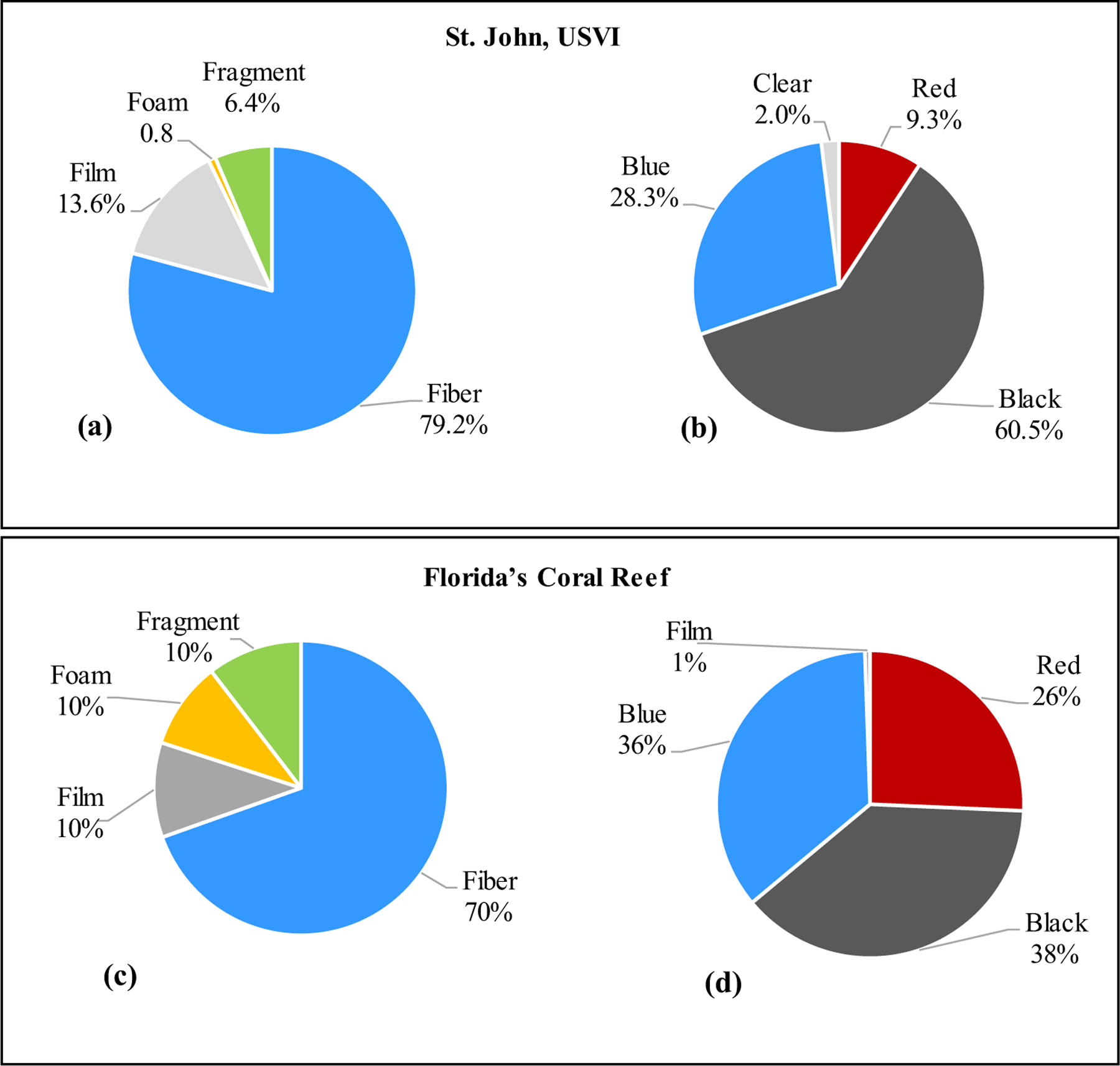
Proportion of various microplastic morphologies and colors found in sub-surface water samples of St. John, U.S. Virgin Islands and Florida’s Coral Reef

All USVI environmental water samples contained MPs with concentrations ranging from 0.03 to 0.28 MP L^−1^ (*n* = 16). The highest and lowest concentrations of MPs observed was at site 6170 and 6129, respectively (Table 2). Fibers were the predominant MP particle morphology found in 79% of the water samples across all sampling stations (Fig. 3a). No fiber bundle, sphere, or pellet MP particles were observed. Black was the prominent MP color observed in USVI environmental water samples at 61% (Fig. 3b).

Microplastics were found in all FL water samples, with concentrations ranging from 0.07 to 0.23 MP L^−1^ (*n* = 12). Site 1288 had the highest concentration of MPs in water with site 1236 having the lowest concentration (Table 3). Fibers were the predominant morphology found in water samples at 70% (Fig. 3c). Colors of MPs in environmental water samples were black (38.3%), blue (35.5%), or red (25.7%) with only one clear fiber found (Fig. 3d).

### Coral tissue samples

**Fig. 4:**
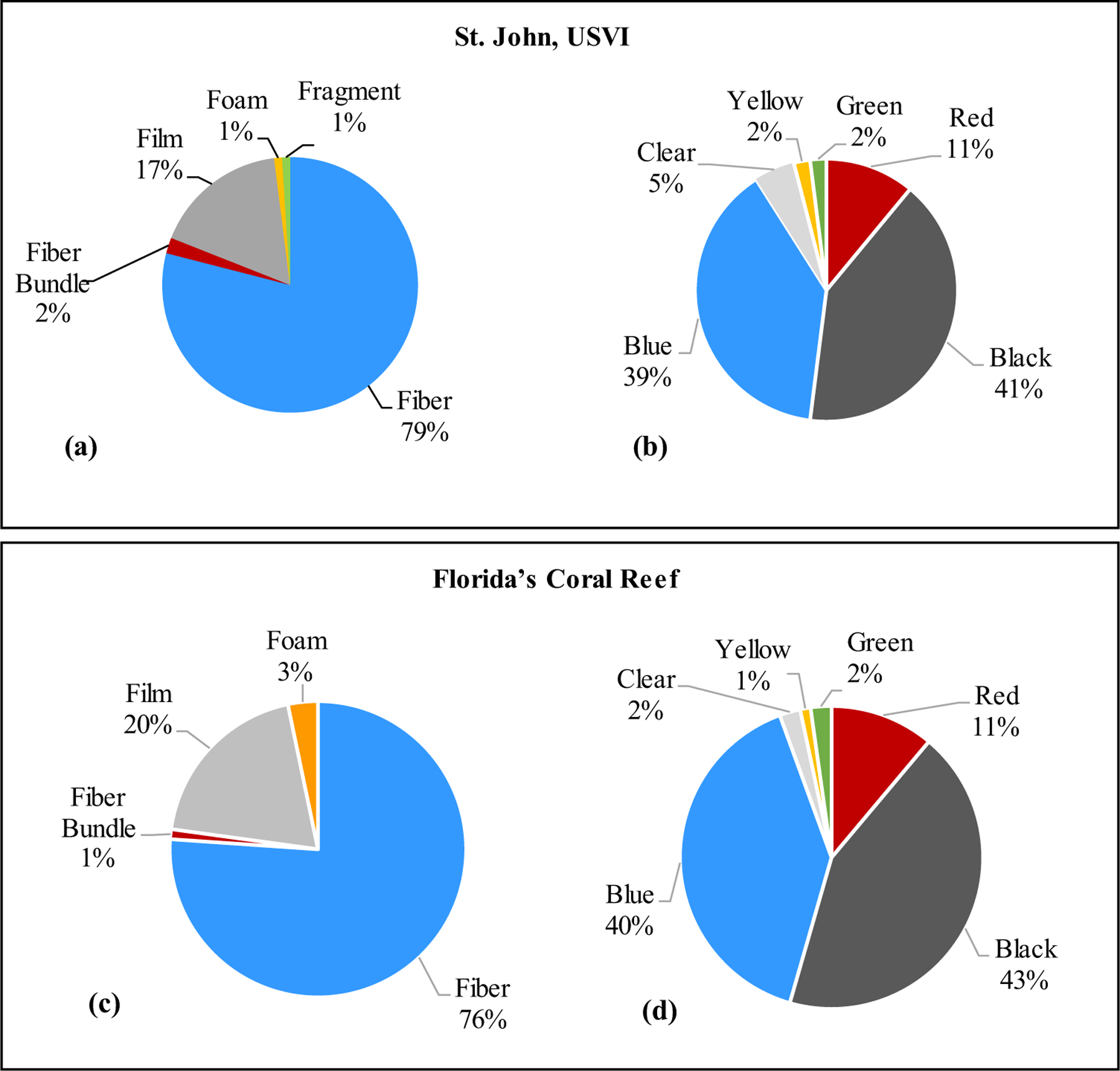
Proportion of microplastic morphologies and color found in coral samples of *Montastraea cavernosa* and *Orbicella faveolata* in St. John, U.S. Virgin Islands and Florida’s Coral Reef

Microplastics were observed in 32 of the 40 coral fragment samples collected from USVI sites and ranged from 0.00 to 0.61 MP cm^−2^ (*n* = 40) from USVI. Mean MP concentrations in tissue averaged per site ranged from 0.04 to 0.36 MP cm^−2^ (*n* = 10) from USVI (Table 2). The highest concentration of 0.36 cm^−2^ coral tissue was found at site 6148, and the site with the lowest concentration of 0.04 cm^−2^ coral tissue was 6210. Collectively, microplastics found in USVI coral tissue consisted of 79% fibers (Fig. 4a). No sphere or pellet MP particles were observed. Most MPs in USVI coral tissue samples were either black (41%) or blue (39%) in color (Fig. 4b).

Microplastics were found in all FL coral samples, with concentrations ranging from 0.08 to 1.17 MP cm^−2^ (*n* = 22). Mean MP concentrations in tissue averaged per site ranged from 0.20 to 0.62 MP cm^−2^ (*n* = 7) (Table 3). Across both species, site 1319 had the highest mean concentration of MP in coral tissue with the lowest concentration found at site 1393. Collectively, MPs found in FL coral tissue consisted of 76% fibers (Fig. 4c). No sphere, pellets, or fragments were observed in coral tissue. The majority of MP in coral was black (43%) or blue (40%) (Fig. 4d).

No significant differences were observed in MP concentrations between corals species from the combined locations (Linear mixed model F_(59)_ = 0.32, *p* = 0.57); therefore, tissue concentrations were averaged for each station for all subsequent analyses.

### Polymer identification of microplastics

**Fig. 5:**
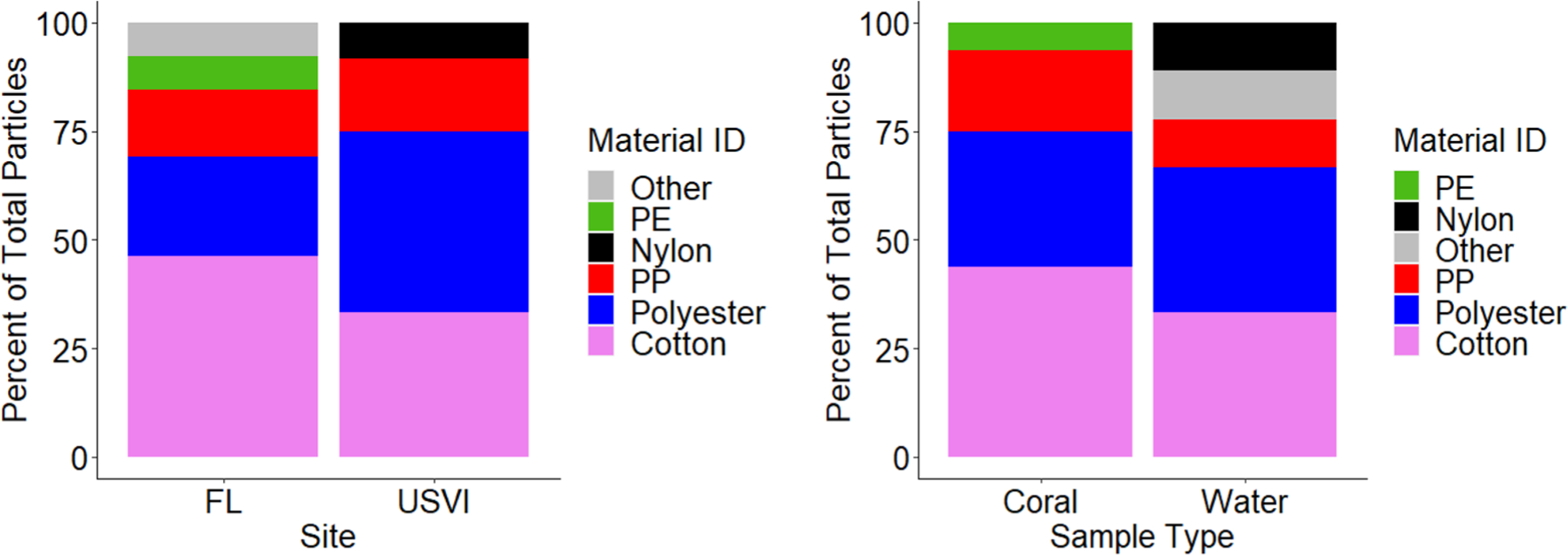
Percent polymer type of particles found in representative subsamples from water and coral samples from St. John, U.S. Virgin Islands and Florida’s Coral Reef. PE = polyethylene, PP = polypropylene

A range of material types were identified using Raman spectroscopy on a representative subsample of particles (*n* = 30), with an average spectral match percentage of 71.3%. The identified materials were polyester, cotton, nylon, polypropylene (PP), and polyethylene (PE) (Fig. 5). Polyester and PP were detected in both coral and water samples from USVI and FL. Fibers and fragments were seen in the blank samples. Fibers constituted most of the particles at 80% with fragments at 20%—all were black and less < 50 μm. The blank samples which contained black fibers may have been from degradation of the metal bands of the mason jars. As none of our representative samples were identified as metal and the fibers were generally less than 50 μm, it is unlikely that particles from contamination were included in our visual counts. Additionally, if there was uncertainty regarding a suspected MP particle it was not counted.

### Coral reef attributes

All DEMO acquired attributes were similar between locations while LPI acquired attributes differed for non-tolerant coral cover (NTCC), non-tolerant coral richness (NTCR), and percent *Orbicella* cover (POC) (Wilcoxon, W_(17)_ = 62.5, *p* = 0.02; W_(17)_ = 61.5, *p* = 0.03; W_(17)_ = 59.5, *p* = 0.03, respectively) (Table 4, SI-C). Full BCG attributes for individual sites and summaries for locations are supplied in Supplemental Information (SI-C and SI-D, respectively).

### Data analysis: microplastics and coral reef attributes

No significant differences were observed in MP concentrations for either coral tissue or water between locations. Furthermore, we failed to detect relationships between MPs in coral tissue and MPs in water or water depth (Liner Mixed Model, t_(17)_ = −1.1, *p* = 0.3; t_(17)_ = − 0.9, *p* = 0.4). However, MPs in coral tissue showed significant positive relationships with rugosity (R), coral density (CD), and percent coral cover (PCC), (Liner Mixed Model, t_(17)_ = 3.6, *p* < 0.01; t_(17)_ = 5.6, *p* < 0.01, t_(17)_ = 5.07, *p* < 0.01, respectively).

**Fig. 6:**
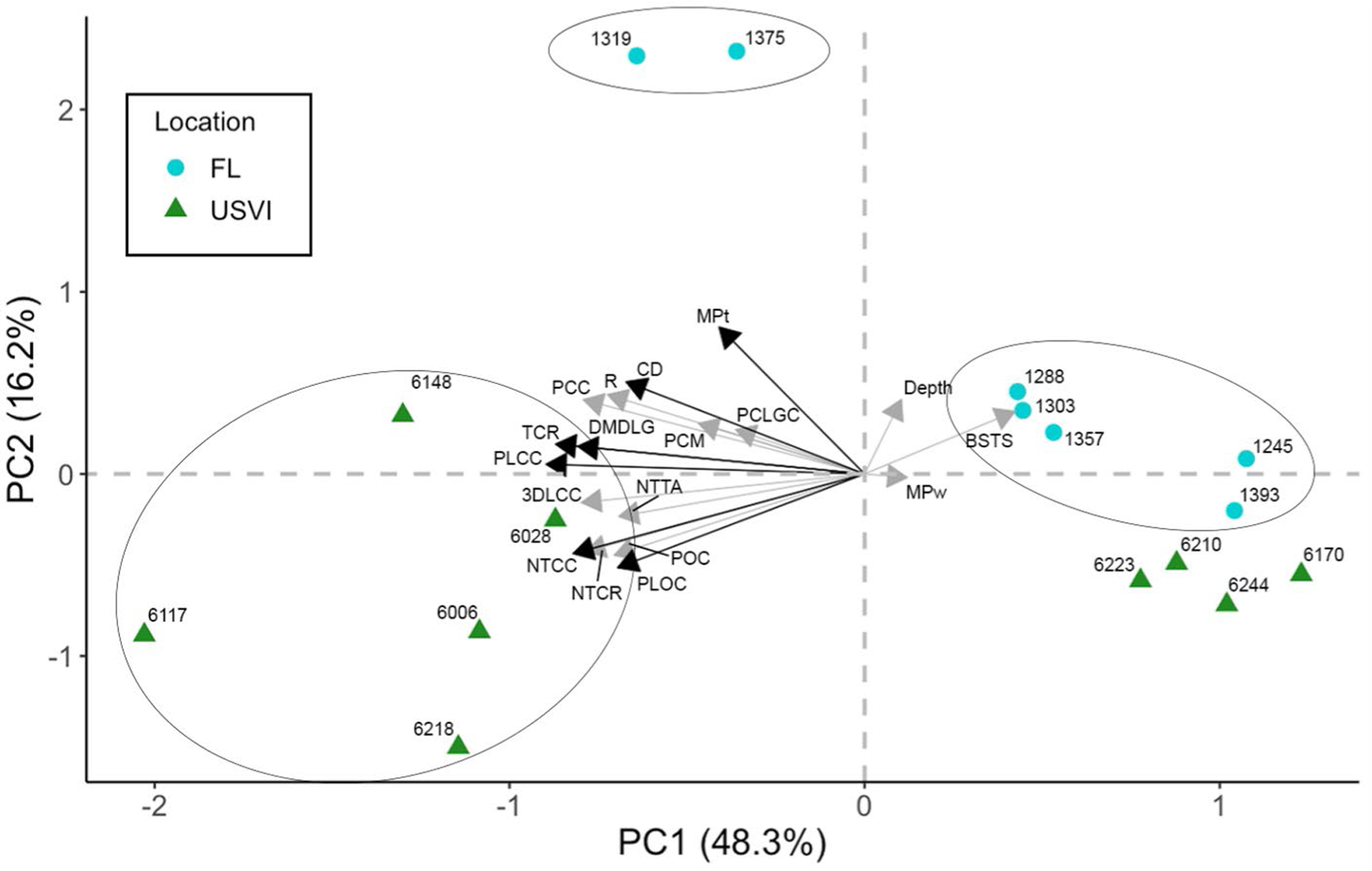
Principal Component Analysis (PCA)(centered and scaled) for all reef attributes and microplastics data for sites in Florida’s Coral Reef (FL; points) and US Virgin Islands (USVI; triangles). Numbers represent station locations. Black vectors represent variables with the greatest influence on the PCA (PCA1 loading scores > 0.8/ <—0.8 and PCA2 loading scores > 0.5/ < −0.5)

Excluding site 6188, the Principal Component Analysis (PCA) split sites into two main groups based on BCG attributes and MP data, with two FL stations isolated in a third cluster (Fig. 6). The USVI sites were generally divided into those that more closely resembling Florida’s Coral Reef and sites that resemble each other, predominantly driven by high values for reef attribute measures like coral density and presence of non-tolerant taxa and low values for bare substrate & turf with sand. Concentrations of MPs in water were not important to the PCA whereas MP concentrations in coral tissue likely contributed to the two FL sites that did not cluster with the others.

**Fig. 7:**
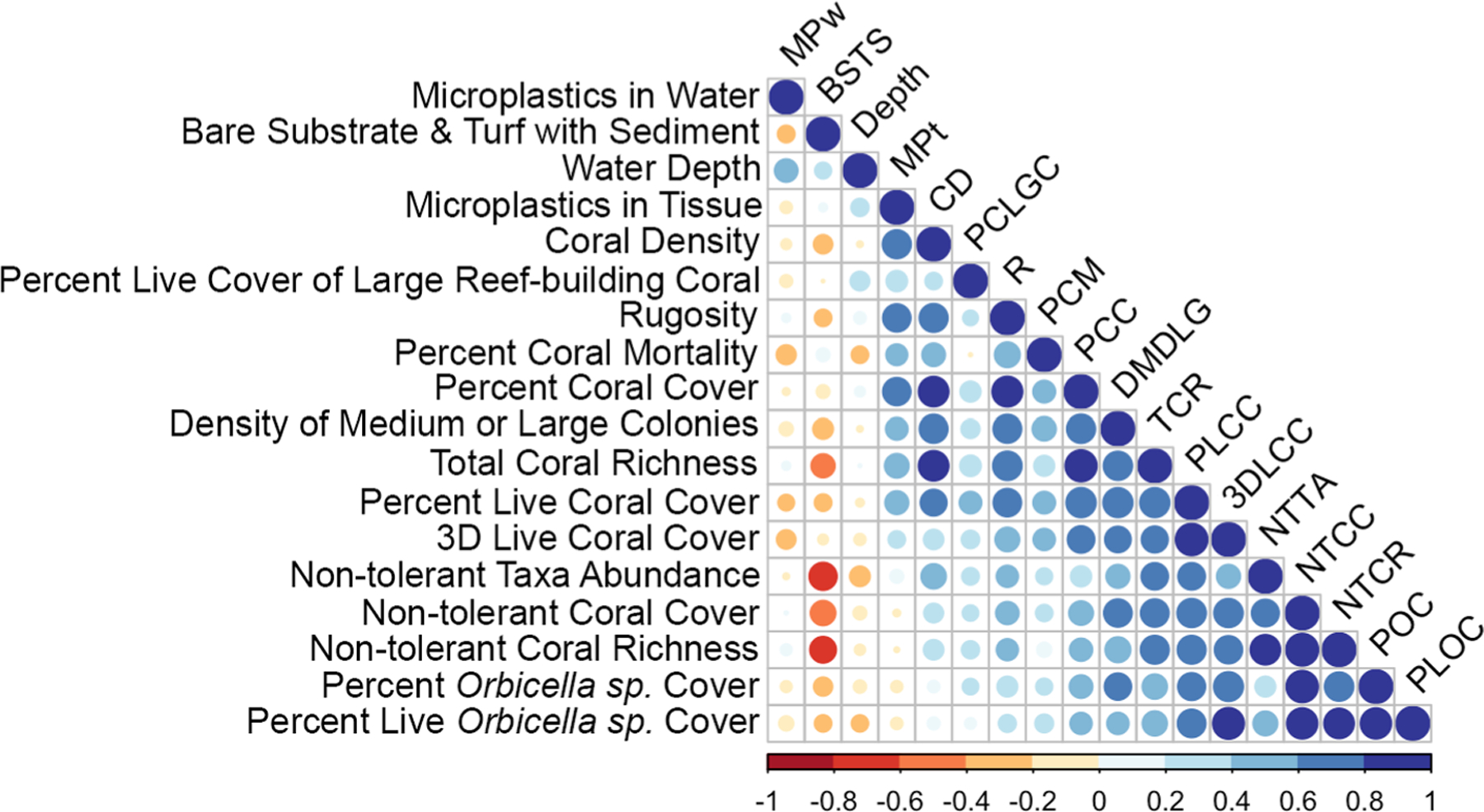
Correlation matrix of relationships between coral reef attributes and microplastics concentrations in water and coral samples. Color and size of points represent Spearman correlation coefficient values with red indicating negative correlations, blue indicating positive correlations, and size representing strength of the relationship. Points displayed based on hierarchical clustering

Several BCG attributes were highly correlated with each other (Fig. 7). In particular, non-tolerant coral cover was positively correlated with non-tolerant coral richness (*r* = 0.94, *p* < 0.001) as expected, and also percent *Orbicella* cover (*r* = 0.91, *p* < 0.001). Microplastics in tissue was strongly correlated with coral density (*r* = 0.75, *p* < 0.01), rugosity (*r* = 0.77, *p* < 0.01), and percent coral cover (*r* = 0.68, *p* < 0.01). Measures of non-tolerant taxa abundance and non-tolerant coral richness were negatively correlated with bare substrate and turf with sediment (*r* = − 0.51, *p* = 0.04 and *r* = − 0.55, *p* = 0.03).

## Discussion

This study is the first to document MPs in coral tissues from either the U.S. Virgin Islands or Florida’s Coral Reef and to explore how MPs in water or tissue relate to coral reef attributes. We quantified MPs in sub-surface water and coral around St. John, USVI and Florida’s Coral Reef; with a representative subset of MPs further analyzed for polymer identification. Using NOAA’s NCRMP LPI and DEMO protocols, we utilized attributes from BCG’s expert panel of coral ecologists, and explored relationships between MPs and coral reef attributes, finding higher MP coral tissue concentrations with greater coral density and percent cover. Microplastics were observed in water at both locations and present in nearly all coral tissue samples demonstrating the ubiquity of MPs in the marine environment and importance of studies that examine their impacts to coral reef habitats.

Though MPs were detected in all water samples, no relationship was observed between any BCG attributes. This is not surprising as MPs in water are both spatially and temporally variable, and there is difficulty in predicting fate and transport (GESAMP 2015; Bigdeli et al. 2022). The life cycle of MPs in the marine environment is dependent on multiple factors including physical properties of the MP (size, shape, density), biofouling, water currents, wind, and Stokes drift (Ballent et al. 2013; Kooi et al. 2017; Li et al. 2020). Thus, the sub-surface water samples collected in this study were, in essence, a snapshot in time in the MPs’ dynamic transport (Imhof et al. 2017). Sediments, however, are believed to be a sink for MPs (Martin et al. 2020; de Smit et al. 2021), making benthic organisms, such as scleractinian coral particularly vulnerable to MPs. Thus, sediments might better represent MP exposure at a particular location than water and deserves further study.

Concentrations of MPs in coral tissue are a function of ingestion and egestion and is not generally well understood. Scleractinian corals are capable of ingesting MPs, as has been documented by multiple studies (Hall et al. 2015; Allen et al. 2017; Hankins et al. 2018; Axworthy et al. 2019; Corona et al. 2020; Grillo et al. 2021; Hankins et al. 2021; Reichert et al. 2024). Only a handful of these studies have documented egestion but in all cases, corals retained only a portion (< 34%) of what they ingested (Allen et al. 2017; Hankins et al. 2018, 2021; Reichert et al. 2018, 2024). The concentrations at which MPs in coral tissue result in a biological response that it is measurable by coral reef condition is unknown, though—if MPs are affecting coral, it would be anticipated that coral reef conditions are poor. Unexpectedly, we observed that as MP concentrations in coral tissue increased, reef attributes were better as we found positive correlations with rugosity, coral density, percent live coral cover, and total coral richness. These relationships could partially be explained by hydrodynamics in coral reefs. A laboratory study with 3D printed coral colonies showed that the ability of a reef to trap MPs increased with canopy density (Mendrik et al. 2024). Another study showed similar trapping efficiencies with high coral density in addition to showing no differences in trapping efficiency among live coral, coral skeleton, or skeletons with a smooth surface (waxed) (Yen et al. 2024). Indeed, a concentration-dependent risk assessment suggest that corals are unlikely affected by environmentally relevant MP concentrations but biofouling and toxicity of MPs may still be a concern (Reichert et al. 2024). Our field-based results suggest higher rugosity, coral density, percent live coral cover, and total coral richness have a positive relationship with MPs found in coral tissue. It should be noted, however, that stressors such as disease or bleaching, which plague Caribbean and Florida reefs (Muller et al. 2020; Brandt et al. 2021; Eakin et al. 2022), were not attributes of the BCG – health conditions that may not be reflected in measures of rugosity, coral density, coral cover, or richness. Moreover, ingestion and retention of MPs in coral vary depending on species (Hankins et al. 2021; Reichert et al. 2024); the corals sampled in this study may not represent the more sensitive coral species to MPs. Mechanistic and toxicology studies are needed to better understand processes of entrapment, ingestion, and egestion of MPs by coral and coral reef assemblages. Monitoring more coral species and health conditions in relation to MP concentrations may also better reflect MP effects on coral reefs.

The general separation of sites between USVI and FL from BCG attributes seen in the principal component analysis are congruent with NCRMP report cards that listed USVI reefs in fair condition (Blondeau et al. 2020) and Florida’s Coral Reef listed as impaired (Towle et al. 2020). Data from NCRMP’s LPI and DEMO protocols are not intended to characterize individual sites but to inform NOAA’s status on coral reefs within a region (Grove et al. 2023; Viehman et al. 2023); we used data from these protocols to quantify coral reef BCG attributes. When used to evaluate impacts of individual stressors, like MPs, there is potential for targeted resource management by identifying direct linkages to stressors affecting the reef assemblage of a specific site. Although MPs in water were not a good predictor of MPs—in coral tissue, we observed positive relationships between MPs and BCG attributes generated from two LPI measures (rugosity and percent *Orbicella* cover) and one DEMO measure (coral density), indicating some NCRMP parameters may more adequately represent MP exposure than others. However, further assessments and analysis are needed with larger datasets to determine if similar relationships are seen in other reef habitats for these reef attributes.

Precise MP sources are often difficult to identify, with each sampling location vulnerable to a unique input of possible pollution sources according to their geography and proximity to human influence (Auta et al. 2017). Macrosized marine debris, such as from litter or improperly managed marine recreation or industry materials (i.e., marine rope, traps, buoys, etc.), are sources of MPs as they degrade in the environment (Thompson 2015). Previous research has also identified atmospheric deposition, stormwater runoff from land, sediment-bound pollution transport, and effluent from waste infrastructure as major sources of MPs into marine systems (Horton & Dixon 2018; Malli et al. 2022). Textile laundering can release up to ~ 700,000 microfibers into wastewater per laundry cycle, or approximately 0.1% of total textile mass per wash (De Falco et al. 2019; Cesa et al. 2020). Though a small proportion of these microfibers evade capture during sewage processing at wastewater treatment facilities (WWTFs) (between 1 and 5%) (Carr et al. 2016), the result may lead to substantial MP deposition into marine areas. Our observations found fibers as the dominant morphology in both USVI and FL samples and supports other studies that found microfibers as the dominate MP morphology in coral reefs (Ding et al. 2019; Jeyasanta et al. 2020; Oldenburg et al. 2021; Tang et al. 2018; Lei et al. 2021; Carbery et al. 2022; Lim et al. 2022; Patterson et al. 2022; Zhou et al. 2022). As such, WWTFs also present a potential target for reducing MP loads in coral reef habitats.

The inclusion of polymer identification for MPs provides chemical evidence that the particle in question is of anthropogenic origin and not naturally occurring. However, it requires precision instruments and a high level of expertise (Primpke et al. 2020) which can be limiting factors to microplastic research (Wiggin et al. 2019; Patti et al. 2020). Polymer identification of a subset of representative samples can be useful as an exploratory effort to determine if additional quantification is necessary. In the current study, polymer identification of our visually quantified MPs resulted in 100% agreement for anthropogenic particles. We did not count particles under 50 μm as it is difficult to accurately determine material visually, and if there was uncertainty regarding a particular MP it was not counted. As such, our values are conservative as we likely underestimated concentrations.

It is estimated that 3% of the plastic produced globally (approximately 12 million tons) enters marine environments on an annual basis (Jambeck et al. 2015; Boucher et al. 2020; Plastics Europe 2021). To protect coral reefs more effectively from MPs, we must better understand exposure pathways and impacts from that exposure. We detected relationships between MPs in coral tissue and BCG attributes, yet mechanistic studies that explore how MPs in tissue affect coral at the individual level and scaled up to the reef level are still needed, regarding both physical and chemical effects. Polymer identification of MPs was important for confirming particles were anthropogenic and is a recommended step. Additionally, measuring MP concentrations in sediment, rather than coral tissue or water, could be a more suitable predictor of microplastic exposure and warrants further study. Expanded datasets, including coral health and models of how MPs impact coral is essential for effective management decisions that better protect coral reefs.

## Supplementary Material

Supplement 1

The online version contains supplementary material available at https://doi.org/10.1007/s00338-024-02596-4.

## Figures and Tables

**Table 1:** Attributes of the coral reef Biological Condition Gradient informed by the National Coral Reef Monitoring Program’s (NCRMP) line point intercept (LPI) and coral demographic (DEMO) protocols

Coral Condition Attribute	Abbreviation	NCRMP Protocol
3D live cover	3DLCC	DEMO
Coral density	CD	DEMO
Density of medium or large colonies	DMDLG	DEMO
Non-tolerant taxa* abundance	NTTA	DEMO
Percent live coral cover	PLCC	DEMO
Percent live cover of large, reef building coral species	PCLGC	DEMO
Percent live *Orbicella* cover	PLOC	DEMO
Percent coral mortality	PCM	DEMO
Bare substrate & turf algae with sediment	BSTS	LPI
Non-tolerant coral* cover	NTCC	LPI
Non-tolerant coral* richness	NTCR	LPI
Percent coral cover	PCC	LPI
Percent *Orbicella* cover	POC	LPI
Total coral richness	TCR	LPI
Weighted rugosity	R	LPI

* See SI-B for non-tolerant taxa

**Table 2:** Mean microplastic (MP) concentrations and (standard deviation) from environmental water and coral tissue samples from St. John, USVI in 2017

Site no	Water (MP L^−1^) (*n* = 16)	Coral (*n* = 10)	
		Mean MP cm^−2^ tissue per site coral	Coral fragments per site
6006	0.19	0.28 (0.15)	4
6025	0.19	n/a	n/a
6028	0.19	0.31 (0.27)	4
6108	0.04	n/a	n/a
6109	0.06	n/a	n/a
6117	0.19	0.22 (0.19)	4
6129	0.03	n/a	n/a
6148	0.14	0.36 (0.18)	4
6170	0.28	0.05 (0.07)	2
6188	0.09	0.15 (0.10)	4
6190	0.12	0.23 (0.20)	3
6210	0.16	0.04 (0.04)	4
6218	0.08	0.15 (0.20)	4
6223	0.15	n/a	n/a
6241	0.07	n/a	n/a
6244	0.08	0.13 (0.00)	2

n/a indicates sites where *Montastraea cavernosa* and *Orbicella faveolata* colonies were not present. See Fig. 1 for site locations

**Table 3:** Mean microplastic (MP) concentrations and (standard deviation) from environmental water and coral tissue samples from Florida’s Coral Reef in 2018

Site no	Water (MP L^−1^) (*n* = 16)	Coral (*n* = 7 sites)	
		Mean MP cm^−2^ coral tissue per site	Coral fragments per site
1236	0.07	n/a	n/a
1244	0.1	n/a	n/a
1245	0.15	0.25 (0.24)	2
1288	0.23	0.32 (0.17)	4
1303	0.12	0.21 (0.15)	4
1319	0.10	0.62 (0.49)	4
1357	0.19	0.26 (0.04)	4
1375	0.18	0.52	1
1384	0.14	n/a	n/a
1387	0.08	n/a	n/a
1390	0.09	n/a	n/a
1393	0.16	0.20 (0.03)	2

n/a indicates sites where *Montastraea cavernosa* and *Orbicella faveolata* colonies were not present. See Fig. 2 for site locations

**Table 4:** Mean and standard deviation of biological condition gradient attributes with significant differences for U.S. Virgin Islands (USVI) and Florida’s Coral Reef (FL)

Location	Non-Tolerant Coral Cover (NTCC)	Non-Tolerant Coral Richness (NTCR)	Percent *Orbicella* Cover (POC)
USVI	4.00 ± 3.92*	1.91 ± 1.58*	1.73 ± 2.53*
FL	0.29 ± 0.49	0.29 ± 0.49	0.00

Asterisk (*) indicates significantly greater than the other location (*p* < 0.05, Wilcoxon)

## Data Availability

The datasets generated during and/or analyzed during the current study are available at ncei.noaa.gov/access/meta-data/landing-page/bin/iso?id=gov.noaa.nodc:0176081, ncei.noaa.gov/archive/accession/0208322, and data.gov.
